# Supplementary material for: Facile synthesis of reduced graphene oxide by modified Hummer's method as anode material for Li-, Na- and K-ion secondary batteries
Source: R Soc Open Sci. 2019 Apr 24;6(4):181978. doi: 10.1098/rsos.181978 (PMC6502357; doi:10.1098/rsos.181978)
Supplement: Supplementary Information [file rsos181978supp1.docx]

Supplementary Information

Facile synthesis of reduced Graphene oxide by modified Hummer’s Method as anode material for Li, Na, and K-ion secondary batteries

Jeonggeun Jo^a^, Seulgi Lee^a^, Jihyeon Gim^a†^, Jinju Song^a††^, Sungjin Kim^a^, Vinod Mathew^a^, Muhammad Hilmy Alfaruqi^a^, Seokhun Kim^a^, Jinsub Lim^b^ and Jaekook Kim^a*^

*a. Department of Materials Science and Engineering, Chonnam National University, 300 Yongbong-dong, Bukgu, Gwangju 61186, Republic of Korea.*

*b. Korea Institute of Industrial Technology(KITECH), Buk-gu, Gwangju 61012, South Korea*

**Fig. S1.** CV result for the prepared rGO samples versus Li metal anode.
